# How Not to Live

**Published:** 1869-01

**Authors:** 


### HOW NOT TO LIVE.

We number among our friends a gentleman who is now nearly seventy years of age; one who, in his peculiar state of health, represents a very large class of people, individual cases of which are within almost every one's knowledge, and in describing him we shall draw a portrait which almost every one will recognize as that of Mr. So-and-so of their own particular acquaintance.

Mr. C—— is a man of a very nervous-bilious temperament, who inherits a predisposition to long life. Of New England origin and a poor boy, he began business behind the counter of a dry-goods store. By the closest attention to his daily work, and a total neglect of the laws of life and health, breathing a vitiated store-atmosphere during the day and an equally poisonous one in his bed-room during the night; eating his meals whenever it was convenient, instead of at regular hours; usually carrying in his pockets a supply of peanuts, candies, or something of that sort, which he would eat from time to time; he became of course an early victim to dyspepsia.

He had hardly arrived at man's estate when he suffered everything from this disease; but instead of endeavoring to cure himself by any sensible course of living, he took to quackery and continued on in his usual habits of life. The consequence has been, that he has long since run the gauntlet of quack medicines, having gobbled down more or less of every nostrum that was ever invented; and what is really remarkable is, that he still lives. The tenacity to life which he inherits from his parents has enabled him to resist the consequences of taking quack medicines *ad libitum.*

Though in advanced life, he still gets down to his business before any of his clerks in the morning and is the last to leave at night. He continues to fill his pockets, as of old, with nuts or other food, and takes his lunch sometimes at one hour, sometimes at another, and his neglect of all the well-known laws of life and health is as persistent as it was when he was a boy of seventeen! He thinks he knows more than all the doctors in general matters, and especially about his own case.

It is perfectly safe to say that he has not known what it is to enjoy a day of tolerable health for forty years! and for twenty-five years his system has not been able to perform its regular functions without the use of artificial means, and is liable at any hour to fall into a bodily condition from the fatal effects of which no human skill could save him.

It often happens that a man becomes infatuated with a certain mode of life, or with certain habits of eating or drinking, or exercise or sleep, and to these he attributes whatever of healthful conditions he may enjoy; but before deciding whether these modes or habits are certainly promotive of good health, it would be safe to propose a few cross-questions and to have an affirmative answer to the following inquiries:

Are you in perfectly good health in all respects? As to the case above, the man inherited a good constitution; in addition to that he lived a very active life, much of it in outdoor exercise, and it was these conditions which kept him alive in spite of his great imprudence.

It is not wise to attribute the inestimable blessing of good health to any one particular observance or habit; nor on the other hand is it well to attribute ill health to any one cause, nor to consider a return to good health as the result of the last thing done. The tendency of a bad cold, if let alone and not renewed, is to cure itself at the end of two weeks, while the last of the dozen or two things taken, or the last doctor called in, gets all the credit, when it should have been given to nature herself.

In many cases persons lose flesh, become weak, lose their ambition, become dispirited, and lose their appetites and energy, when along comes some old man or Indian chief or hoary hag, and advises a tea made of half a dozen different kinds of roots, leaves, barks, and flowers; the patient takes it and begins to get well right away, and is in perfect ecstacies; he prescribes it with great confidence to every second man he meets, whatever may be the nature of his friend's affliction, whether it be apoplexy, cancer, consumption, or hole in the pocket, and it will not unfrequently be found that at the time of beginning to take the compound, some striking change occurred in the state of the weather or season, or some new and exhilarating employment was engaged in, or he fell heir to a fortune, or got married, or went to sea, or undertook a long journey on foot or on horseback; any one of which things is competent to change the whole current of a man's circulation under favoring circumstances.

One thing is perfectly certain, that a regular systematic and temperate life is promotive of high health and length of days the world over; while irregular eating and sleeping, exercise and indulgence will always, sooner or later, undermine the health, impair the usefulness, efficiency, and enjoyment of the individual, ending in premature disablement and death.

